# Metabolic Changes and Oxidative Stress in Diabetic Kidney Disease

**DOI:** 10.3390/antiox10071143

**Published:** 2021-07-19

**Authors:** Midori Sakashita, Tetsuhiro Tanaka, Reiko Inagi

**Affiliations:** 1 Division of Nephrology and Endocrinology, Graduate School of Medicine, The University of Tokyo, Tokyo 113-8655, Japan; msakashita-tky@umin.ac.jp; 2 Division of CKD Pathophysiology, Graduate School of Medicine, The University of Tokyo, Tokyo 113-8655, Japan; inagi-r@m.u-tokyo.ac.jp

**Keywords:** diabetic kidney disease, oxidative stress, organelle crosstalk, Nrf2, bardoxolone methyl

## Abstract

Diabetic kidney disease (DKD) is a major cause of end-stage kidney disease, and it is crucial to understand the pathophysiology of DKD. The control of blood glucose levels by various glucose-lowering drugs, the common use of inhibitors of the renin–angiotensin system, and the aging of patients with diabetes can alter the disease course of DKD. Moreover, metabolic changes and associated atherosclerosis play a major role in the etiology of DKD. The pathophysiology of DKD is largely attributed to the disruption of various cellular stress responses due to metabolic changes, especially an increase in oxidative stress. Therefore, many antioxidants have been studied as therapeutic agents. Recently, it has been found that NRF2, a master regulator of oxidative stress, plays a major role in the pathogenesis of DKD and bardoxolone methyl, an activator of NRF2, has attracted attention as a drug that increases the estimated glomerular filtration rate in patients with DKD. This review outlines the altered stress responses of cellular organelles in DKD, their involvement in the pathogenesis of DKD, and discusses strategies for developing therapeutic agents, especially bardoxolone methyl.

## 1. Introduction

Diabetic kidney disease (DKD) is one of the most important chronic complications of diabetes, has a significant impact on the prognosis of patients by increasing the incidence of cardiovascular complications, and may lead to end-stage kidney disease (ESKD).

Although the overall rate of DKD is decreasing with improvements in glycemic control, the number of patients with DKD is increasing in proportion to that with diabetes [1]. Furthermore, the incidence of DKD in the elderly and number of cases exhibiting the pathology of nephrosclerosis is increasing. Therefore, there is an urgent need to elucidate the pathogenesis of DKD, prevent its progression, and explore novel therapeutic approaches.

Conventionally, diabetic nephropathy has been considered to follow a typical progression, especially in type 1 diabetes mellitus. The process begins with glomerular hyperfiltration progressing to microalbuminuria, macroalbuminuria, and subsequent decrease in the glomerular filtration rate (GFR), eventually leading to ESKD. In recent years, however, a growing number of cases of chronic kidney disease (CKD) associated with diabetes mellitus, in which GFR decreases without albuminuria, has been recognized as a new subgroup of DKD [2]. This form of progression is thought to be due to the aging of the diabetic population and the widespread use of angiotensin-converting enzyme (ACE) inhibitors and angiotensin II receptor blockers (ARBs), which suppress intraglomerular pressure. Many of these subgroups show a pathology of nephrosclerosis and are expected to be strongly associated with atherosclerosis.

The two mainstays of treatment are the control of blood pressure and blood glucose levels and the use of ACE inhibitors and ARBs to control proteinuria and slow the progression of DKD. Recently, a multifaceted approach that includes dyslipidemia and hypertension has been proposed to ameliorate the damage caused to the kidneys [3]; but currently, there is no specific therapeutic agent for DKD. In addition, the number of patients with ESKD due to DKD is increasing because patients with DKD experience a faster estimated GFR (eGFR) decline than those with chronic kidney disease of other causes. Therefore, the development of new drugs that are specific to DKD is long-awaited.

In diabetes mellitus the function of intracellular organelles such as mitochondria is altered even before the increase in blood glucose levels, leading to changes in systemic metabolism. Various cellular stress responses associated with metabolic changes are involved in the development and progression of DKD, including oxidative stress, endoplasmic reticulum (ER) stress, advanced glycation end products (AGEs), inflammation, cytokines, growth factors, epigenetic mechanisms, and histone and chromosomal abnormalities. Although these mechanisms have been studied, the overall etiology of DKD remains unclear [4].

This review outlines the involvement of renal metabolic alterations, organelle dysfunction, and cellular stress responses, especially oxidative stress, in the pathogenesis of DKD. We will also discuss potential therapeutic approaches for DKD, in particular the potential renoprotective effects of bardoxolone methyl.

## 2. Energy Metabolism and Mitochondrial Function in the Kidneys

A healthy kidney is composed of many different types of cells, and the proper functioning of each cell type is important for the overall function of the kidney. Different cell types have different demands for energy and oxygen, with the proximal tubular cells and thick ascending loop of Henle (TAL), the center of active transport, using most of the oxygen consumed by the kidney to produce adenosine triphosphate (ATP).

The kidneys require a large amount of energy and thus have the second highest density of mitochondria after the heart. Most of the energy is supplied by ATP, which is mainly obtained through oxidative phosphorylation. Oxidative phosphorylation occurs in the mitochondria by conversion of the energy from fuels such as sugars and lipids into high-energy chemical bonds. It plays a central role in aerobic metabolism and can produce energy more efficiently in the presence of sufficient oxygen compared to anaerobic metabolism by processes such as glycolysis. Aerobic metabolism, including oxidative phosphorylation, can produce around 30 mol of ATP from 1 mol of glucose and around 100 mol from 1 mol of palmitic acid, whereas glycolysis can produce only 2 mol of ATP from 1 mol of glucose.

The main substrates used by each cell for energy production differ and are determined by the role and location of each cell. Cells in the proximal tubule, TAL, and distal convoluted tubule, where ATP consumption is high to actively reabsorb sodium, glucose, and other metabolites filtered into the urine, utilize fatty acids and ketones to produce ATP, while cells in the glomerulus and collecting ducts preferentially utilize glucose [5].

The selectivity of the substrate for such energy production is important for the maintenance of normal function. For example, the expression of rate-limiting enzymes of the glycolytic system is maintained at low levels in proximal tubular cells for efficient reabsorption of glucose by sodium-glucose linked transporters (SGLTs). Furthermore, a recent study using genome-wide transcripts from a large cohort revealed that the kidneys of patients with CKD and fibrosis exhibit reduced expression of genes involved in fatty acid metabolism and accumulation of oil droplets [6]. A metabolomic study of patients with diabetes also demonstrated the importance of energy metabolism in the pathogenesis of DKD. This study compared the kidneys of patients with diabetes affected for more than 50 years, differing only in the presence or absence of nephropathy, and found elevated levels of proteins involved in glucose metabolism and antioxidant activity in patients without nephropathy [7]. This study identified that the glycolytic system, especially pyruvate kinase M2 in podocytes, plays a major role in this protective effect. Another metabolomic study that compared urine metabolites between patients with diabetes with and without DKD showed a significant difference in water-soluble organic anions. This suggests that mitochondrial metabolism may greatly contribute to the progression of DKD [8].

In recent years, the cardio- and renoprotective effects of SGLT2 inhibitors have been attracting attention, and kidney metabolism may also play a major role in the renoprotective effects of these therapeutic agents. Recent studies have shown that the increase in ketone bodies after SGLT2 inhibitor administration may have renoprotective effects by inhibiting the detrimental effects of mechanistic target of rapamycin complex 1 (mTORC1) in podocytes [9].

Furthermore, Falkevall et al. showed VEGF-B, which regulate endothelial fatty acid transport in the endothelium, plays a major role in the accumulation of lipid droplets in the podocyte and the increase in albuminuria in mice. They also found that VEGF-B level correlates with DKD advancement in humans [10].

In the proximal tubules, the substrates of the TCA cycle are fat and amino acids and they produce a large amount of ATP. In the early stage of diabetes, the TCA cycle is enhanced in response to hyperfiltration, but in the late stage, it does not work sufficiently and cell function is reduced [11].

As we have discussed, the hyperglycemic environment augments the glycolytic system in podocytes and alters the TCA cycle in the tubules. These diabetes-induced metabolic changes induce mitochondrial dysfunction, ER stress, and other organelle responses [12], which will be discussed in the next chapters.

## 3. ROS Production and Mitochondrial Dysfunction

Mitochondria are the centers of aerobic metabolism. During oxidative phosphorylation, hydrogen ions captured in the form of nicotinamide adenine dinucleotide (NADH) and flavin adenine dinucleotide (FADH_2_) by glycolysis and tricarboxylic acid cycles pass through a series of redox carriers (complexes I–IV) in the mitochondrial cristae, sequentially lowering their energy levels and passing them to their final acceptor, oxygen (O_2_), to become water (H_2_O). Complexes I–IV are called the electron transport chain because electrons are exchanged between enzymes and coenzymes at the inner mitochondrial membrane. In complexes I, III, and IV, hydrogen ions are pumped out of the mitochondrial matrix into the intermembrane space, creating a concentration gradient of hydrogen ions across the inner mitochondrial membrane. Finally, adenosine diphosphate (ADP) is phosphorylated to ATP using the hydrogen concentration gradient.

During this electron transport chain, reactive oxygen species (ROS) are produced. When electrons leak prematurely out of the electron transport chain of complexes I and III, O_2_ is partially reduced to produce superoxide (O_2_^●−^). This superoxide is rapidly decomposed into hydrogen peroxide (H_2_O_2_) by superoxide dismutase (SOD). Superoxide and H_2_O_2_ are highly reactive, and some of them are used for cellular signaling, but in excess, they modify lipids, proteins, and nucleic acids and disrupt their normal functioning. Therefore, to maintain a certain concentration, they are degraded by enzymes to become inactive substances. Dysregulation of ROS is well known in DKD and will be discussed later.

Mitochondria not only play a pivotal role in energy and ROS production, but also regulate many cellular processes, including cell proliferation, differentiation, cell death, (apoptosis and necrosis), inflammation, and adaptation. Mitochondria are also deeply involved in tissue damage and repair. Since some of these mitochondrial functions are compromised in DKD and ROS are produced in these processes, repairing mitochondrial functions can be targeted for therapy. DKD disrupts the functions necessary for normal mitochondrial function, including mitochondrial biogenesis, fission, and fusion [13].

Dynamin related protein-1 (DRP1) is recruited from the cytoplasm, phosphorylated, and plays an essential role in mitochondrial fission and mitochondrial fragmentation occurs in DKD [14,15], *Drp1* knockout in podocytes was shown to prevent the progression of DKD in mice [16]. In addition, preventing DRP1 phosphorylation reduces mitochondrial fragmentation and improves DKD [17]. Similarly, pharmacological inhibition of Drp1 resulted in the improvement of DKD [16,18].

Mitophagy occurs via the PTEN-induced kinase 1 (PINK1)-parkin or mitophagy receptor pathway. In the former pathway, PINK1 accumulates on the outer mitochondrial membrane in damaged mitochondria. Parkin, a cytoplasmic ubiquitin E3 ligase, is recruited by PINK1 and activates E3 ligase, ultimately causing mitophagy. In the latter pathway, BCL2/adenovirus E1B 19 kDa protein-interacting protein 3 (BNIP3), BNIP3-like (BNIP3L)/NIX, or FUN14 domain containing 1 (FUNDC1) in the mitochondrial outer membrane is directly bridged to the autophagosome via LC3B.

The expression of PINK1 and parkin is decreased in DKD, indicating that mitophagy is impaired [16,19]. The expression of optineurin, which is required for mitophagosome formation, is decreased in the tubular cells of patients with diabetes [20]. Conversely, increased expression of optineurin in glucose-loaded tubular cells ameliorates cellular senescence, accumulation of ROS, and activation of NLR family pyrin domain containing 3 (NLRP3) inflammasomes [21].

In DKD, mitophagy is impaired in podocytes and PINK1 expression is reduced in the podocytes of streptozotocin (STZ)-treated mice [22]. Furthermore, forkhead Box O1 (FOXO1) expression in podocytes restores PINK1 expression and alleviates STZ-induced DKD [23]. Progranulin regulates mitophagy by activating the SIRT1-peroxisome proliferator-activated receptor-γ coactivator-1α (PGC-1α)-FOXO1 pathway, but its expression is reduced in diabetes [24].

Mitochondrial biogenesis is also important and PGC-1α is heavily involved in this process. PGC1α has been shown to contribute to recovery from kidney injury via de novo NAD production from amino acids [25].

DKD causes mitochondrial damage, including increased mitochondrial fission and decreased mitochondrial biogenesis or fusion. Future research is necessary to determine how DKD-induced damage to these mitochondria affects disease progression, but damaged mitochondria release ROS or cause metabolic abnormalities, making them potential targets for therapy. In one study, when only the mitochondrial DNA was recombined into different strains of mice, the metabolic activities of various organs, including their life span, reflected the properties of the recombined mitochondria. The mitochondrial differences between the species compared in this study are comparable to racial differences in humans, demonstrating the importance of mitochondrial differences in biological activity [26].

## 4. ER Stress and ROS Production

The ER plays an important role in protein expression, folding, and glycosylation. When protein misfolding increases, the unfolded protein response (UPR) pathway is initiated, and this response to ER stress plays an important role in DKD through increasing urinary albumin excretion and the induction of inflammation [27]. ER stress has been implicated in lipid dysregulation and obesity and lipotoxicity is known to be involved in the development of diabetes mellitus [28].

This UPR pathway is activated by one of three major ER stress sensors: pancreatic eukaryotic translation initiation factor-2α (eIF-2α) kinase (PERK), inositol-requiring protein (IRE1), or activating transcription factor6 (ATF6). The ER chaperone protein GRP78/binding immunoglobulin protein (BiP) plays a central role in controlling the UPR pathway. In the resting phase, BiP binds to the transmembrane sensors PERK, IRE1, and ATF6. When ER stress occurs, BiP binds to misfolded proteins and dissociates with ER stress sensors, activating these proteins. The IRE1α/X-box-binding protein (XBP-1) pathway is evolutionarily conserved and IRE1α dissociated from BiP activates XBP-1. This activated XBP-1 induces the transcription of UPR-associated genes, such as PERK and ATF4. Another ER stress sensor PERK phosphorylates eIF-2α during ER stress. Phosphorylated eIF2α inhibits general protein translation and facilitates the translation of specific proteins, ATF4 and nephrin. ATF4 enables the transcription of UPR target genes, such as C/EBP homologous protein (CHOP) and TRB3. CHOP is thought to work mainly as an inducer of apoptosis. Lastly, when ER stress occurs, ATF6 is transported to the Golgi apparatus and cleaved by site 1 and site 2 proteases.

ER stress is involved in DKD pathology in some cultured cell lines and the UPR pathway is activated by hyperglycemia, hyperlipidemia, and AGEs. Mice with STZ-induced diabetes showed increased levels of ER stress and enhanced apoptosis in glomerular and tubular cells [29]. Diabetic CHOP deficient mice are known to show less proteinuria compared to wild-type mice [30]. In addition, STZ-induced diabetes in Trb3 knockout mice resulted in elevated urinary albumin and increased mRNA expression of inflammatory cytokines and chemokines in the renal cortex compared to wild-type mice, despite similar levels of blood glucose [31]. Diabetes selectively inhibits the nuclear translocation of XBP1 in podocytes, induces ATF6 and CHOP, and exacerbates DKD [32].

## 5. Oxidative Stress in DKD

Oxidative stress can be classified into two major categories: ROS and reactive nitrogen species; the former includes peroxides, superoxide, and hydroxyl radicals and is largely responsible for kidney disease.

ROS are mainly produced in the mitochondria, especially in the electron transport chain. In addition to mitochondria, low levels of ROS are produced in the ER and peroxisomes by several enzymatic reactions, including xanthine oxidase, uncoupled nitric oxide synthase, and NAD(P)H oxidase (NOX). In the kidneys, mitochondria and the NOX family are the primary sources of endogenous ROS.

In mitochondria, ROS are degraded by SOD2 in the mitochondrial matrix and SOD1 in the mitochondrial intermembrane space. Catalase and glutathione peroxidase are also important for ROS detoxification. Catalase, located in peroxisomes next to the mitochondria, reacts with H_2_O_2_ to catalyze the production of H_2_O and O_2_. Glutathione peroxidase reduces H_2_O_2_ by transferring the energy of the reactive peroxide to a small sulfur-containing protein called glutathione. Peroxiredoxins also degrade H_2_O_2_ in the mitochondria, cytoplasm, and nucleus.

NOXs are another important source of ROS [33]. NOXs are inactivated under normal physiological conditions. However, in disease conditions such as hypertension and diabetes, its transcription and translation are activated, or the enzyme itself becomes more active. Among the seven isoforms of NOXs, including NOX1–5, DUOX1, and DUOX2, NOX4 is the major isoform in the kidneys and has been extensively studied in DKD. NOX4 expression increases during excess production of ROS and presence of high glucose levels and contributes to glomerular hypertrophy and mesangial expansion.

ROS are intrinsic to cellular function and are present at low and constant levels in healthy cells. For example, tubular feedback has a major impact on the pathogenesis of DKD and ROS produced by NOX4 and 2 are involved in this regulation [34]. Superoxide anions promote tubular feedback by tightening the afferent arterioles and scavenging nitric oxide in the macula densa. However, ROS can oxidize and modify some cellular components, causing irreversible damage to DNA and preventing cells from functioning properly. This implies that ROS have a dual role as a toxic factor and a protective or signaling factor. In fact, it is known that the production of ROS is reduced in mice treated with STZ, a well-known diabetic model. The role of ROS depends on whether the timing and location are proper, and whether the balance between production and degradation is appropriate. In other words, oxidative stress arises from both unregulated ROS production and inadequate ROS removal by antioxidant systems.

The response to oxidative stress is also related to other stress responses that occur in DKD, such as AGE and hypoxia. Chronic hyperglycemia leads to the formation of AGEs, which bind to AGE receptors and induce oxidative stress. AGEs are formed in the late stages of non-enzymatic glycation of proteins and sugars (Maillard reaction). Aldehydes and ketones react with the lysine and *N*-terminus of proteins, and these react with proteins inside and outside the cell via reactive intermediates such as α-dicarbonyl compounds and oxoaldehyde to form AGEs.

Glucose is the least reactive and a stable substance and these reactions do not occur under normal conditions. On the other hand, AGEs such as carboxymethyllysine, carboxyethyllysine, and pentosidine are formed following the polyol pathway, which converts excess glucose into sorbitol in diabetes. In the non-oxidative pathway, AGEs are generated from methylglyoxal formed by anaerobic glycolysis and 3-deoxyglucosone formed by the Amadori rearrangement. These AGEs cause additional reactions in a receptor-independent and receptor for AGE (RAGE)-mediated manner. In the former, AGEs directly react with the basement membrane and extracellular matrix, affecting membrane permeability, thermostability, and membrane potential. The latter leads to the activation of ROS and nuclear factor kappa B (NF-κB) through various signaling pathways. RAGE is present in the tubules, mesangium, podocytes, blood vessels, and nerves in humans [35].

There is an interaction between AGEs and ROS, and it has been reported that ROS can irreversibly cross-link AGE-induced changes in collagen. Conversely, it has been reported that AGEs reduce the expression of antioxidant enzymes such as SOD, glutathione levels, and increase the production of ROS itself.

## 6. Oxidative Stress and Hypoxia

Hypoxia is another important mechanism in the pathophysiology of DKD. The kidneys are physiologically hypoxic, but this condition is known to be exacerbated in DKD. The hypoxia-inducible factor (HIF), the master regulator of the hypoxic response, is activated by the administration of HIF-prolyl hydroxylase (HIF-PH) inhibitors [36,37].

The use of HIF-PH inhibitors has been shown to improve metabolism and has been shown to reduce various renal dysfunction [38,39] and improved glycogen metabolism that resulted in reduced acute kidney injury [40]. In the STZ-induced diabetes model, the HIF-PH inhibitor treatment has also been shown to increase glycolytic activity and improved the disease course of DKD [41]. Furthermore, the leptin deficient diabetic black and tan brachyury (BTBR) *ob*/*ob* mice treated with HIF-PH inhibitors showed decreased body weight, lower blood glucose levels, improved insulin sensitivity, and better lipid profiles [42].

Furthermore, there is an interaction between hypoxia and oxidative stress. Increased oxidative stress leads to increased intracellular oxygen consumption, resulting in progressive hypoxia. Administration of indoxyl sulfate, a representative uremic toxin, to the isolated proximal tubules of rats and humans has been shown to induce hypoxia via induction of oxidative stress [43]. Conversely, it has been proposed that oxidative stress is induced under hypoxia, a seemingly paradoxical phenomenon in which mitochondria act as sensors of hypoxia and promote the production of ROS [44].

## 7. Organelle Crosstalk and Interplay of the Organelle Stress

As we discussed, diabetes-induced metabolic changes result in organelle stresses, such as mitochondrial stress including oxidative stress and increased mitochondrial fission and ER stress. Each organelle is interconnected and, in recent years, direct contact between organelles has attracted attention.

The region of ER that is in contact with mitochondria is called the mitochondria-associated ER membrane (MAM). They were firstly identified as a fraction that is enriched in proteins for lipid synthesis and trafficking. Defects of MAMs caused by ER stress, abnormal lipid metabolism, or autophagy can lead to mitochondrial damage via Ca^2+^ influx. Recent progress of imaging technologies enables visualizing MAMs with confocal and lattice light-sheet microscopy [45,46]. Overexpression of mitofusin 2, which is essential for MAM organization, has been shown to activate lipid-inducible pathways in the liver and to act on mitochondrial fission. Although there are few studies on MAM in the kidney, MAM also contributes to the activation of NLRP3 [47], a known component of inflammasomes, and the involvement of NLRP3 in DKD has also been shown [21,48]. Thus, disorders of organelles, especially those in ER and mitochondria, affect each other and alter cellular functions.

**Figure 1:**
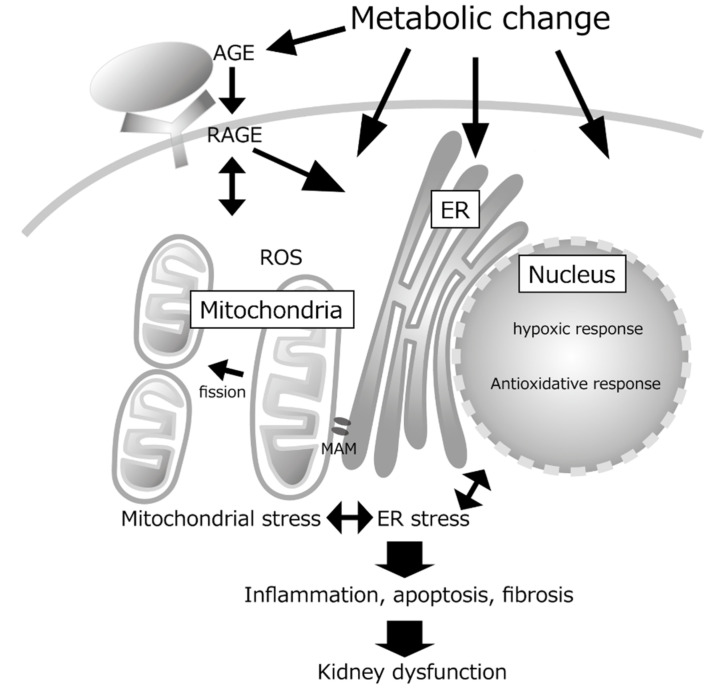
Organelle crosstalk and interconnected stress response. Metabolic changes in DKD induces organelle stresses including mitochondrial stress and endoplasmic reticulum (ER) stress. These organelle stress together with advanced glycation end products (AGEs) cause inflammation, apoptosis, and fibrosis, resulting in kidney dysfunction.

Furthermore, these stress responses are interconnected with AGEs generated from metabolites and interstitial hypoxia (Figure 1).

## 8. NRF2 as a Master Regulator of Antioxidative Stress

Oxidative stress is involved in the stress response of various organelles induced by DKD and NRF2 is a master regulator of the response to oxidative stress. NRF2 is a transcription factor that maintains homeostasis and typical target genes of Nrf2, including NAD(P)H quinone reductase, antioxidative enzymes such as glutathione S-transferase, glutathione synthase, heme oxygenase 1, thioredoxin, SOD, and catalase, and their involvement in oxidative stress defense has been described in previous chapters.

**Figure 2:**
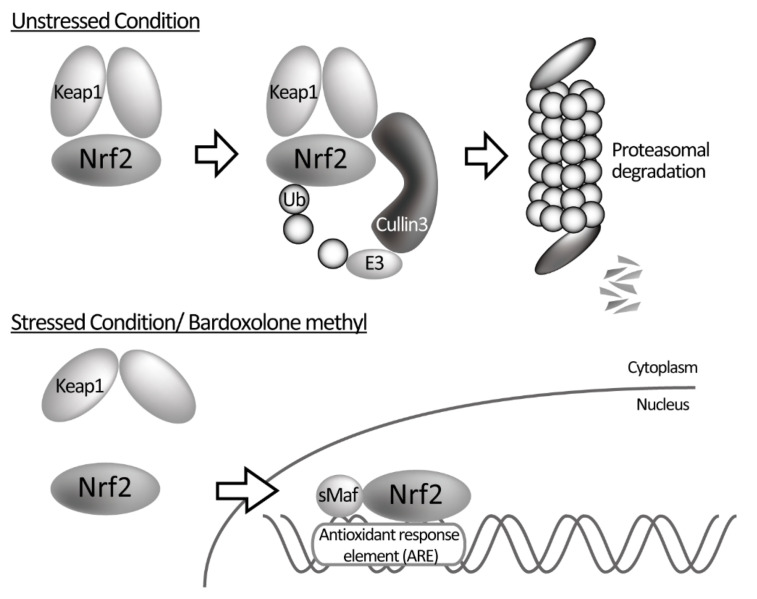
Regulation of NRF2. Under normal conditions, NRF2 is captured by KEAP1, ubiquitinated by Cullin3-type E3 ubiquitin ligase, and degraded by the proteasome. Under oxidative stress or bardoxolone methyl administration, NRF2 evades from degradation due to the conformational change of KEAP1 and is transferred into the nucleus. NRF2 binds to the antioxidant response element (ARE) along with the small musculoskeletal fibrosarcoma oncogene homologue (sMaf), resulting in the expression of downstream genes.

NRF2 is produced at a constant rate and its concentration is strictly controlled by the following mechanism to enable a rapid response to oxidative stress. In the absence of oxidative stress, NRF2 is captured in the cytoplasm by the Kelch-like erythroid cell-derived protein with CNC homology-associated protein 1 (Keap1), ubiquitinated by the Cullin-type E3 ubiquitin ligase, and degraded by the proteasome to maintain a constant low concentration. KEAP1, which plays a major role in regulating the concentration of NRF2, contains several highly reactive cysteine residues. When oxidative stress or electrophilic substances react with the cysteine residues of KEAP1 and cause a conformational change in KEAP 1, its affinity to NRF2 is reduced. This allows NRF2 to evade degradation and moves into the nucleus, leading to a rapid increase in the concentration of NRF2 in the nucleus. There, NRF2 forms a complex with a small musculoaponeurotic fibrosarcoma oncogene homolog, binds to the antioxidant response element (ARE) in the DNA sequence, and regulates the expression of more than 250 antioxidant and anti-inflammatory genes (Figure 2). In macrophages, NRF2 directly suppresses inflammatory cytokines at the transcriptional level [49]. Many NRF2 activators, including bardoxolone methyl, dissociate KEAP 1 from NRF2 by altering the structure of KEAP 1, allowing NRF2 to move into the nucleus. This is thought to be particularly effective in DKD, where oxidative stress and increased inflammation are thought to be involved in its progression [50,51].

Studies have shown that NRF2 is important for organ protection and increased NRF2 activity may extend the lifespan of the patient. The DNA-binding activity of NRF2 in the nuclear extracts of the liver was positively correlated with the average lifespan of about ten rodent species, including the house mouse with a 30-year lifespan, whereas the KEAP1 protein content was negatively correlated to lifespan. Furthermore, progerin, a mutation of lamin A that covers the nuclear membrane and the causative protein of a premature aging syndrome called the Hutchinson–Gilford syndrome, is known to inhibit the binding of NRF2 to ARE sequences in the nucleus and reduce the expression of genes downstream of NRF2 [52].

## 9. NRF2 Function in the Kidneys

Our understanding of the function of NRF2 has been greatly enhanced by the generation of knockout mice. *Nrf2* knockout mice develop normally and show no obvious abnormalities at a young age [53,54], but female knockout mice of the ICR strain develop lupus-like autoimmune nephritis after 60 weeks of age, resulting in decreased renal function and worse prognosis [55]. In addition, the survival rate of *Nrf2* knockout mice was shown to decrease in lipopolysaccharide-induced sepsis [56,57], smoking-related lung injury [58,59], and acetaminophen-induced liver injury models [59,60,61], indicating that NRF2 has a protective effect on multiple organs. From these results, it can be expected that Keap1 knockout mice would have increased longevity due to increased NRF2 activity. However, all *Keap1* knockout mice died within 21 days of birth due to hyperkeratosis of the esophagus [62]. Thus, *Keap1* conditional knockout mice have been developed [61,63] and the effects of increased NRF2 activity or NRF2-activating drugs have been investigated.

In addition to the age-related nephritis described above, *Nrf2* knockout mice showed significant renal dysfunction and deterioration of renal tissue in various models including diabetic [64], ischemia–reperfusion [65], cisplatin-induced nephropathy [66], and lupus nephritis models [67]. Table 1 shows the main results of studies showing the role of NRF2 in animal models of kidney diseases. In the ischemia–reperfusion model, the expression of downstream genes of *Nrf2* is known to be increased [68] and *Nrf2* knockout mice showed marked tubular damage, whereas mice with increased NRF2 activity by Keap1 knockdown showed a noticeable improvement in tubular damage. The downstream genes of *Nrf2* are mainly expressed in the tubules and suppress renal injury in the early stage of reperfusion by suppressing glycolysis and the citric acid cycle and increasing the expression of many genes, including those encoding antioxidants such as glutathione and *Nadph* [66]. In addition, T-cell specific activation of *Nrf2* suppressed creatinine elevation in an ischemia–reperfusion model [69].

A DKD study showed that levels of urinary 8-hydroxy-2’-deoxyguanosine, an indicator of oxidative stress, are higher in patients with diabetes compared to the control and correlate with other indicators of complications such as proteinuria [70]. In addition, the expression of NRF2 is elevated in the renal tissue of patients with type 2 diabetes [70]. In a STZ-induced diabetic animal model, oxidative stress was shown to further increase in *Nrf2* knockout mice and marked renal injury compared with *Nrf2^+/+^* mice [64,71] and the *Nrf2* activator, such as sulforaphane or cinnamic aldehyde, ameliorated kidney function only in *Nrf2^+/+^* mice. In addition, the amount of white adipose tissue was markedly decreased in *Nrf2* knockout mice, while *Nrf2* knockout mice with the db/db background showed further lipid abnormalities. These results indicate that NRF2 has a protective effect on pancreatic beta cells [72] and improves insulin resistance in diabetes mellitus.

## 10. Oxidative Stress as a Therapeutic Target

Antioxidants have been shown to have renoprotective effects in animal studies but have not shown significant effects in clinical trials. This may be due to the removal of oxidative stress to a degree that is physiologically necessary. For example, preischemic preconditioning acts renoprotectively during renal ischemia via ROS, but this protective effect is lost when antioxidants are administered [86].

Removal of impaired or dysfunctional mitochondria is also a possible approach. mTOR inhibitors promote autophagy and remove impaired mitochondria, but may also inhibit cell proliferation, making clinical application difficult [87]. Furthermore, chronic use of mTOR inhibitors may contribute to glucose intolerance and lead to dysfunction of the kidneys.

Metformin is one of the first line treatments in diabetes and its protective effect on organs such as the kidney is mediated by mitochondrial complex I inhibition and AMPK activation [88,89].

Thiazolidinediones are ligands for peroxisome proliferator-activated receptor-γ (PPARγ), activate PGC1α, contribute to the PPARγ-independent activation of 5’ AMP-activated protein kinase signaling, and have been reported to have renoprotective effects. On the other hand, a direct relationship between mitochondrial biogenesis and renoprotective effects has not been shown.

Bardoxolone methyl (also known as RTA-402 or 2-cyano-3,12-dioxoolean-1,9-dien-28-oic acid [CDDO]-Me) is a C30 compound, a synthetic triterpenoid with an oleic acid skeleton. These CDDO compounds inhibit inflammatory substances such as nitric oxide synthesis and cyclooxygenase 2. Initially, bardoxolone methyl was developed as a therapeutic agent for malignancy, as its administration to experimental animals suppressed tumor growth in prostate, pancreatic, and estrogen receptor-negative breast cancers along with other cancers, and its growth inhibitory effect was also suggested in pancreatic, rectal, and ovarian cancers, glioblastoma, and neuroblastoma [90]. The mechanism of action of bardoxolone methyl on tumor suppression is thought to be due to the suppression of the NF-κB pathway in addition to NRF2 activation. In a clinical trial for malignant tumors, bardoxolone methyl-treated patients showed a decrease in serum creatinine [91] and thus came into light as a treatment for CKD.

The mechanism by which bardoxolone methyl improves the renal function in response to DKD has not been fully elucidated, but it is thought that its antioxidant effects via activation of the transcription factor NRF2 plays a major role. Previous studies have shown that bardoxolone methyl suppresses mesangial cell contraction by inhibiting calcium influx into mesangial cells [92] and preserves vascular endothelial cell function by increasing the nitric oxide level [93]. It is also known that bardoxolone produces toxic metabolites in rodents, making it difficult to evaluate the effects of bardoxolone [94]. Long-term experimental results using cynomolgus monkeys showed increase in the expression of Nrf2 downstream genes, elevated eGFR and increased urinary protein, but there were no deleterious findings of hyperfiltration in the histology of the kidneys [85].

## 11. Results of Previous Clinical Trials or Bardoxolone Methyl for CKD

As mentioned above, bardoxolone methyl was initially developed for the treatment for malignancy, and many clinical trials have since been conducted (Table 2). During a phase I study in patients with malignant tumors, creatinine decreased and eGFR increased in patients with impaired renal function [91]. Subsequently, a phase II study was conducted in 18 patients with CKD and diabetes mellitus with an eGFR of 30 mL/min/1.73 m^2^ and the safety of the drug was confirmed [95].

One of these was the BEAM study [96], a randomized, double-blind, placebo-controlled trial conducted on patients with type 2 diabetes mellitus and CKD with an eGFR of 20–45 mL/min/1.73 m^2^. A total of 127 adult patients treated with ACE inhibitors or ARBs for at least 8 weeks were randomly assigned to receive placebo or 25, 75, or 150 mg of bardoxolone methyl (each taken once daily). In the bardoxolone methyl group, GFR increased from 4 weeks after the start of treatment, peaked at 12 weeks, and persisted until 52 weeks. The change in eGFR at 24 weeks, the primary endpoint, was significantly higher in the bardoxolone methyl group than in the placebo group, with a mean change of approximately 10 mL/min/1.73 m^2^ in both groups. This increase in eGFR was maintained at 52 weeks, which was the secondary endpoint. In contrast, albuminuria increased at 24 and 52 weeks. The most frequent adverse event in the bardoxolone methyl group was muscle spasm, which occurred in approximately half of the patients but was mild. In addition, hypomagnesemia, mild elevation of alanine aminotransferase, and gastrointestinal disturbances were more frequent in the bardoxolone methyl group than those in the placebo group, suggesting that bardoxolone methyl may be a promising treatment for DKD.

Based on these results, the BEACON trial was conducted [97], which was a multicenter, randomized, double-blind, placebo-controlled study of patients with type 2 diabetes and CKD in the eGFR range of 15–30 mL/min/1.73 m^2^. The primary endpoint was the occurrence of ESKD or death due to cardiovascular events (the difference in the dose of bardoxolone methyl was due to the formulation; the 20 mg dose in this study was equivalent to the 75 mg dose in the BEAM study). However, at a median of 9 months, the composite outcome of cardiovascular events and the incidence of heart failure, which were secondary endpoints, were significantly higher in the bardoxolone methyl group and the study was terminated for safety reasons. There was no statistically significant difference in the primary composite outcome (ESKD or death from cardiovascular events) or the incidence of ESKD. On the other hand, 96 of 1088 patients in the bardoxolone methyl group were hospitalized or died of heart failure, compared with 55 of 1097 patients in the placebo group, which had a significant increase in the hazard ratio of 1.83 (95% confidence interval: 1.32–2.55; *p* < 0.01). The eGFR, blood pressure, and urinary albumin-to-creatinine (ACR) ratio significantly increased and body weight decreased in the bardoxolone methyl group. Risk factors for the development of heart failure in the BEACON trial included a high basal brain natriuretic peptide (BNP) level (>200 pg/mL) and a history of hospitalization for heart failure [98]. It has been suggested that bardoxolone methyl is involved in the expression of endothelin, which is involved in the regulation of fluid volume in the kidneys, and this may have led to salt and water retention and the development of heart failure.

Although there was a temporary suspension due to the discontinuation of the BEACON trial, the TSUBAKI trial, a phase II trial in Japan for patients with stage 3 and 4 CKD, was restarted in December 2014 and completed in September 2017. The study included CKD patients with type 2 diabetes (15 ≤ eGFR < 60 mL/min/1.73 m^2^) who were administered ACE inhibitors or ARBs, the G3 group that included CKD stage G3 patients with ACR < 300 mg/gCr, and the G4 group that included CKD stage G4 patients with ACR < 2000 mg/gCr. Patients were assigned to the placebo and bardoxolone groups. In the bardoxolone group, patients received 5–15 mg of bardoxolone methyl once daily for 16 weeks to evaluate safety and efficacy. Patients with type 1 diabetes, BNP levels > 200 ng/mL, or a history of heart failure were excluded. In the G3 group, GFR was measured by inulin clearance to exclude the possibility that bardoxolone methyl affects creatinine excretion. As a result, GFR in the bardoxolone methyl group increased by 6.64 mL/min/1.73 m^2^ after 16 weeks of treatment compared to that at the beginning of the study [99]. A phase III study of bardoxolone methyl in patients with diabetes and CKD stage G3 and G4 DKD (AYAME study; ClinicalTrials.gov number: NCT02316821) is currently underway. The primary endpoint of the study was a 30% or greater decrease in eGFR from baseline or the first occurrence of ESKD.

The effect of bardoxolone methyl is also evaluated in various renal diseases other than DKD. The phase II/III CARDINAL trial for Alport syndrome and phase II PHOENIX trial for a rare cause of CKD have been completed and the phase III FALCON trial and phase III EAGLE trial for ADPKD and the phase II/III MERIN trial for rapidly progressive renal disease are ongoing to evaluate the effect of bardoxolone methyl on renal function.

In DKD, the increase in GFR with bardoxolone methyl may be effective and its potential for improving renal function is attractive. However, bardoxolone methyl increases albuminuria [84,100] and the presence of albuminuria is considered an indicator of renal prognosis, cardiovascular events, and life expectancy in CKD [101]. Previous studies have also shown that the chronic overload of albumin endocytosis triggers oxidative stress and ER stress, leading to renal injury [102,103]. On the contrary, the increase in urinary albumin with bardoxolone methyl is proportional to the increase in eGFR in clinical trials [84,100], and it is thought to be due to changes in urinary protein uptake by tubular megalin and not due to renal tissue degeneration in experiments with bardoxolone methyl in cynomolgus monkeys [85]. Therefore, the long-term effects of bardoxolone methyl on GFR and the increase in albuminuria should be closely monitored. At the same time, the renoprotective effect of bardoxolone methyl in other renal diseases, such as Alport syndrome, should also be carefully evaluated to determine whether it improves long-term prognosis.

## 12. Conclusions

DKD is a global concern, and the development of specific therapies for the pathogenesis is an urgent need. Metabolic changes in DKD induces oxidative stress and organelle dysfunction. Bardoxolone methyl, an Nrf2 activator might be the potential therapeutics for DKD by improving oxidative stress.

## Figures and Tables

**Table 1:** Function of Nrf2 in the kidney. The following is a list of major studies that have demonstrated the function of Nrf2 in the kidney using animal models. (KO, knockout; CKO, conditional knockout; KD, knockdown; ds-DNA, double- stranded DNA; AGE, advanced glycation end product; CDDO, 2-cyano-3,12-dioxolane-1,9-dien-28-oic acid; BUN, blood urea nitrogen; AST, aspartate transaminase; IRI, ischemia–reperfusion-injury).

Disease Model	Intervension	Results of the Study	Ref.
Aging	*Nrf2*-KO	Increased mortality and worsened renal function were observed in female mice, with lupus nephritis-like findings accompanied by increased spleen weight and increased ds DNA.	[55,73]
Lupus nephritis	*Nrf2*-KO	*Nrf2*-KO mice showed improved renal function and increased survival rate and reduced immune complex deposition in renal tissue	[74]
	*Nrf2*-KO	*Nrf2*-KO mice showed decreased survival, increased spleen weight, increased oxidative stress, and aggravated fibrosis of renal tissue.	[67]
DKD (STZ)	*Nrf2*-KO	*Nrf2*-KO mice showed a similar increase in blood glucose after STZ administration, but decreased creatinine clearance and urinary albumin excretion, worsened renal pathology, and increased AGE, oxidative stress, and fibrosis markers, which were ameliorated by NRF2 activator administration.	[64,71,75]
Bilateral IRI	*Nrf2*-KO	*Nrf2*-KO mice showed elevated creatinine, worsened histology, and marked elevation of cytokines, but prior administration of N-acetylcysteine suppressed the creatinine elevation.	[65]
	CDDO-Imidazole/Bardoxolone methyl	In the CDDO-Im preadministration group, life expectancy, renal function, and renal tissue damage were improved and acute phase inflammatory cytokines were reduced.	[76,77]
Unilateral IRI	*Nrf2*-KO/*Keap1*-KD/*Keap1*-CKO	*Nrf2*-KO mice showed exacerbation of tubular damage and oxidative stress, while *Keap1*-KD and *Keap1*-CKO suppressed creatinine elevation and increased antioxidant markers.	[66]
Sepsis model	LysM-*Keap1*-KO/ LysM-*Nrf2*-KO	LysM-*Keap1*-KO mice showed improved survival and decreased BUN, AST, and inflammatory cytokines, while LysM-*Nrf2*-KO mice showed worsening of these parameters.	[78]
Cisplatin nephropathy	*Nrf2*-KO/ CDDO-Im	*Nrf2*-KO mice showed increased mortality, elevated creatinine, and worsened renal tissue damage, while CDDO-Im administration improved renal tissue.	[65,79,80]
NEP25-induced podocyte injury	*Keap1*-KD	Improved renal tissue, fibrosis markers, and podocyte damage in *Keap1*-KD mice.	[81]
Rhabdomyolysis (myoglobin) nephropathy	-	The expression of downstream genes of *Nrf2* was increased in rhabdomyolysis model induced by glycerol administration; chlormethiazole alleviated these changes.	[82]
5/6 nephrectomy	-	In the 5/6 nephrectomy group, there was a decrease in *Nrf2* expression and an increase in *Keap1* expression.	[83]
Adriamycin, Angiotensin II-induced proteinuria	Keap1-KD	Keap1-KD mice showed increased albuminuria in adriamycin nephropathy, the angiotensin II model, and in the protein overload model.	[84]
Cynomolgus monkeys	bardoxolone methyl	Administration of bardoxolone methyl increased creatinine clearance and urinary albumin; no abnormalities in blood tests or renal tissue were noted after 1 year of treatment. The increase in urinary albumin may be due to decreased megalin expression in the tubules.	[85]

**Table 2:** Results of clinical studies of bardoxolone methyl on diabetic kidney disease.

Phase	Kidney Function	Patient Number	Dose (mg/day)	Duration	ΔeGFR (Average ± S.D.) (mL/min/1.73m^2^)
1st [91]	Ccr ≥ 60 or Cr < 2.0 eGFR < 60 (sub analysis)	36 (10)	5–1300 5–1300	21 days 21 days	26.4% ± 3.2% 35.6% ± 6.8%
2nd [95]	man 1.5 ≤ Cr ≤ 3.0, woman 1.3 ≤ Cr ≤ 3.0	18	25–75	8 weeks	7.2 ± 5.3
2nd (BEAM [96])	20 ≤ eGFR ≤ 45	227	25 25–75 25–100	24/52 weeks	Results in 24 wk/52 wk 8.2 ± 1.5/ 5.8 ± 1.8 11.4 ± 1.5/ 10.5 ± 1.8 10.4 ± 1.5/ 9.3 ± 1.9
3rd (BEACON [97])	15 ≤ eGFR < 30	2185	20	9 months	5.5 ± 0.2
2nd (TSUBAKI [99])	30 ≤ eGFR < 60 ACR < 300, 15 ≤ eGFR < 30 ACR < 2000	120	5–15	16 weeks	6.64
3rd (AYAME)	15 ≤ eGFR < 60, ACR ≤ 3500	(700)	5–15	2–3 years	(ongoing)
